# Factors associated with antibiotic prescribing for acute bronchitis at a university health center

**DOI:** 10.1186/s12879-020-4825-2

**Published:** 2020-02-26

**Authors:** Valerie J. Morley, Emily P. C. Firgens, Rachel R. Vanderbilt, Yanmengqian Zhou, Michelle Zook, Andrew F. Read, Erina L. MacGeorge

**Affiliations:** 1 Center for Infectious Disease Dynamics, Department of Biology, The Pennsylvania State University, University Park, State College, PA USA; 2 Department of Communication Arts & Sciences, The Pennsylvania State University, University Park, State College, PA USA; 3 University Health Services, The Pennsylvania State University, University Park, State College, PA USA; 4 Huck Institutes for the Life Sciences, The Pennsylvania State University, University Park, State College, PA USA; 5 Department of Entomology, The Pennsylvania State University, University Park, State College, PA USA

**Keywords:** Antibiotic stewardship, Antibiotic prescribing, Acute bronchitis, Respiratory tract infections, Student health

## Abstract

**Background:**

Antibiotics are not indicated for treating acute bronchitis cases, yet up to 70% of adult acute bronchitis medical visits in the USA result in an antibiotic prescription. Reducing unnecessary antibiotic prescribing for acute bronchitis is a key antibiotic stewardship goal set forth by the Centers for Disease Control and Prevention. Understanding what factors influence prescribing for bronchitis cases can inform antimicrobial stewardship initiatives. The goal of this study was to identify factors associated with antibiotic prescribing at a high-volume student health center at a large US university. The Pennsylvania State University Health Services offers on-campus medical care to a population of over 40,000 students and receives over 50,000 visits every year.

**Methods:**

We conducted a retrospective chart review of acute bronchitis visits for the 2015–2016 academic year and used a multivariate logistic regression analysis to identify variables associated with antibiotic prescribing.

**Results:**

Findings during lung exams increased the likelihood of an antibiotic prescription (rales OR 13.95, 95% CI 3.31–80.73; rhonchi OR 5.50, 95% CI 3.08–10.00; percussion abnormality OR 13.02, 95% CI 4.00–50.09). Individual clinicians had dramatically different rates of prescribing (OR range 0.03–12.3). Male patients were more likely than female patients to be prescribed antibiotics (OR 1.68, 95% CI 1.17–2.41). Patients who reported longer duration since the onset of symptoms were slightly more likely to receive prescriptions (OR 1.04 per day, 95% CI 1.03–1.06), as were patients who reported worsening symptoms (OR 1.78, 95% CI 1.03–3.10). Visits with diagnoses or symptoms associated with viral infections or allergies were less likely to result in prescriptions (upper respiratory tract infection (URI) diagnosis OR 0.33, 95% CI 0.18–0.58; sneezing OR 0.39, 95% CI 0.17–0.86; vomiting OR 0.31, 95% CI 0.10–0.83). An exam finding of anterior cervical lymphadenopathy was associated with antibiotic prescribing (tender OR 3.85, 95% CI 1.70–8.83; general OR 2.63, 95% CI 1.25–5.54).

**Conclusions:**

Suspicious findings during lung examinations (rales, rhonchi, percussion abnormality) and individual healthcare providers were important factors influencing antibiotic prescribing rates for acute bronchitis visits. Patient gender, worsening symptoms, duration of illness, symptoms associated with viral infections or allergies, and anterior cervical lymphadenopathy also influenced prescribing rates.

## Background

In the United States, 30% of outpatient antibiotic prescribing is estimated to be unnecessary, resulting in almost 47 million unnecessary antibiotic prescriptions each year [1, 2]. Excessive antibiotic prescribing drives the spread of antibiotic resistance, which contributes to increased morbidity, mortality, and economic costs associated with infections [3–5]. In response, the 2015 U.S. National Action Plan for Combatting Antibiotic-Resistant Bacteria set a goal of reducing inappropriate antibiotic prescribing in outpatient settings by 50% by 2020 [6].

A major source of unnecessary outpatient antibiotic prescriptions is acute bronchitis cases [7–10]. Acute bronchitis is a common self-limited respiratory illness, characterized predominantly by cough, typically lasting less than 3 weeks [7, 11]. In the US in 2011, cough was the most common illness-related reason for ambulatory care visits, accounting for 2.6 million outpatient visits [12]. A study in the UK estimated that 44/1000 adults are affected by acute bronchitis each year [13]. Antibiotics are not effective for treating acute bronchitis, which is usually of viral etiology [11], and long-standing professional guidelines recommend against antibiotics for uncomplicated cases [14, 15]. Nevertheless, US adults are prescribed antibiotics for acute bronchitis approximately 60–70% of the time [7–9, 16]. Further, relative to other upper respiratory tract infections for which antibiotic treatment is not indicated (e.g., nasopharyngitis, laryngitis), providers are especially likely to prescribe for acute bronchitis [8, 17–20]. Due to the prevalence of overprescribing, the U.S. Centers for Infectious Disease Control (CDC) has identified acute bronchitis cases as a major opportunity for reducing unnecessary outpatient antibiotic prescribing [21].

Although acute bronchitis presents an opportunity to improve antibiotic stewardship, there is little consensus regarding effective stewardship interventions for ambulatory care [22–24]. A diversity of interventions have been proposed, but evidence supporting their effectiveness remains sparse [23, 24]. Implementation of outpatient stewardship programs could be aided by identifying the factors driving overprescribing, which might point to interventions that target those drivers [22]. Factors driving antibiotic overprescribing may differ between hospital and outpatient settings and could include diagnostic uncertainty, real or perceived patient expectations for antibiotics, time pressures, or gaps in provider knowledge [25, 26].

Identifying drivers of prescribing for acute bronchitis could suggest potential interventions, but relatively few studies have focused on identifying these predictors. Prior studies of upper respiratory tract infection prescribing (including for acute bronchitis) in the USA have shown higher rates of antibiotic prescribing in rural (vs. urban) practices [8, 10], when patients have multiple diagnoses [27] or illness of longer duration [28], when providers are advanced practitioners rather than physicians [9], and when providers experience greater diagnostic uncertainty [27]. Since most studies have utilized data reported to insurance companies or national agencies [13, 16, 23], few previous studies have examined how physical exam findings influence prescribing for acute bronchitis. In the few studies that have included data from patient charts, purulent nasal discharge, purulent sputum, abnormal respiratory exam, tonsillar exudate, and sinus tenderness have been reported to be moderately associated with prescribing [20, 29]. In addition, US prescribing rates for uncomplicated acute bronchitis are higher for younger adults (18–39) than older adults (40+) [16], suggesting that factors influencing bronchitis prescribing for young adults are particularly good targets for evaluation and intervention.

University student health clinics provide an opportunity to study antibiotic prescribing in young adult patient populations. In the US, college students comprise a sizeable cohort of the population, with 20.1 million students enrolled in higher education, including 13.8 million students enrolled at 4-year degree-granting institutions [30]. At these 4-year institutions, there are 165.5 annual visits to student health centers for every 100 enrolled students, 37% of which are for respiratory tract infections [31]. Despite evidence that unnecessary antibiotic prescribing is high in young adult populations [16], antibiotic stewardship programs are almost nonexistent at most student health centers, and best stewardship practices are not yet defined. Understanding what drives unnecessary antibiotic prescribing in student health centers is a first step towards evidence-based stewardship policies in these settings, and findings can also inform stewardship efforts with providers treating young adults in similar contexts (e.g., urgent care clinics).

The goal of this study was to identify patient and visit factors associated with antibiotic prescribing for young adults diagnosed with acute bronchitis at a high-volume student health center at a large US university. We conducted a retrospective chart review of all visits with an acute bronchitis diagnosis for the 2015–2016 academic year at the Pennsylvania State University’s Student Health Center. This work is part of a multi-study interdisciplinary effort to improve antibiotic stewardship in emerging adult populations, with an initial focus on students at residential colleges.

## Methods

### Study site

The Pennsylvania State University Health Services (UHS) offers on-campus medical care to PSU students and their dependents, serving over 40,000 students in more than 50,000 visits yearly. At the time of the study, 28 clinicians saw patients at UHS. During the study period, 21 of these clinicians (9 doctors of medicine (MDs), 2 doctors of osteopathic medicine (DOs), 8 physician assistants (PAs), and 2 nurse practitioners (NPs)) diagnosed at least one patient with acute bronchitis. The remaining clinicians did not diagnose acute bronchitis in the period studied, and therefore they do not appear in the data set.

### Data collection and Curation

UHS staff identified 1451 visits with acute bronchitis diagnoses during the 2015–2016 academic year (August–May). Honest brokers were then employed and trained to access the electronic medical records for these visits, extract deidentified data (data excluding information that could be used to identify individual patients), and enter it in the secure database manager REDCap for use by the researchers. Data extracted included patient characteristics, visit characteristics, symptoms recorded, exam findings, secondary diagnoses, tests ordered, and antibiotic prescriptions (see Table 1). A double-entry procedure was used to provide a reliability check on data extracted from a randomly selected sample (*N* = 69; ~ 5%) of the visits. This check indicated adequate data quality (agreement > 96% across all variables) for the intended analyses; identified discrepancies were corrected [32–34].

**Table 1:** Descriptive statistics (*n* = 1031) and bivariate analysis

Variable	Visit Count (%)	Odds Ratio (95% CI)	Bivariate *p*-value
Date and Time			
visit date	1028 (99.7%)	0.99 (0.99-0.99)	*p* < 0.001 **
week day	1028 (99.7%)	range 0.64-1.28	*p* = 0.63
time of day	1021 (99.0%)	0.99 (0.99-0.99)	*p* = 0.24
Patient Characteristics			
gender			*p* = 0.03*
female (reference group)	636 (61.7%)	-	
male	390 (37.8%)	1.34 (1.02-1.76)	
not recorded	5 (0.5%)	-	
race			*p* = 0.43
white (reference group)	594 (57.6%)	-	
multiple	92 (8.9%)	0.90 (0.55-1.44)	
Asian	50 (4.8%)	0.97 (0.51-1.79)	
black	21 (2.0%)	1.04 (0.38-2.54)	
Hispanic	6 (0.6%)	-	
international	7 (0.7%)	-	
Pacific islander	1 (0.1%)	-	
not recorded	260 (25.2%)	-	
academic status			*p* = 0.27
undergraduate student	932 (90.4%)	-	
graduate student	85 (8.2%)	0.86 (0.52-1.39)	
spouse/dependent	3 (0.3%)	-	
not recorded	11 (1.1%)	-	
height (inches)	1013 (98.2%)	1.04 (1.00-1.08)	*p* = 0.02*
weight (pounds)	1016 (98.5%)	1.00 (0.99-1.01)	*p* = 0.09
Visit Characteristics			
provider	see Fig 3	range 0.05–4.63	*p* < 0.001**
days since onset (patient reported) ††	1016 (98.5%)	1.02 (1.01-1.03)	*p* < 0.001**
severity (patient reported)			*p* = 0.009*
mild (reference group)	61 (5.9%)	-	
moderate	342 (33.2%)	0.48 (0.27-0.86)	
severe	32 (3.1%)	1.13 (0.46-2.70)	
not recorded	596 (57.8%)	-	
progression (patient reported)			*p* < 0.001**
stable/no change (reference group)	274 (26.6%)	-	
worsening	317 (30.7%)	2.27 (1.77-3.77)	
improving	108 (10.5%)	1.06 (0.60-1.84)	
not recorded	332 (32.2%)	-	
antibiotics in past month	40 (3.9%)	0.46 (0.19-1.00)	*p* = 0.07
Additional Diagnosis			
upper respiratory infection	197 (19.1%)	0.26 (0.16-0.39)	*p* < 0.001**
suspicious cough	77 (7.5%)	1.02 (0.61-1.67)	*p* = 0.93
allergic rhinitis	32 (3.1%)	0.51 (0.19-1.17)	*p* = 0.14
fever	17 (1.6%)	2.58 (0.98-6.92)	*p* = 0.05
viral syndrome	13 (1.3%)	-	-
tonsillitis	6 (0.6%)	-	-
influenza	4 (0.4%)	-	-
mononucleosis	3 (0.3%)	-	-
Common Symptoms Recorded†			
throat symptoms			
sore throat	402 (39.0%)	1.42 (1.08-1.85)	*p* = 0.01*
painful swallowing	130 (12.6%)	0.58 (0.37-0.89)	*p *= 0.02*
hoarseness	167 (16.2%)	0.80 (0.55-1.56)	*p* = 0.25
swollen glands in neck	112 (10.9%)	1.08 (0.70-1.63)	*p* = 0.73
systemic symptoms			
headache	244 (23.7%)	0.79 (0.57-1.09)	*p* = 0.15
documented fever	68 (6.6%)	0.70 (0.42-1.17)	*p* = 0.17
fever symptoms (patient reported)	255 (24.7%)	0.94 (0.69-1.28)	*p* = 0.71
chills	147 (14.2%)	0.94 (0.65-1.37)	*p* = 0.73
sweats	159 (15.4%)	1.19 (0.82-1.75)	*p* = 0.36
nasal symptoms			
stuffy nose	665 (64.5%)	0.86 (0.66-1.14)	*p* = 0.29
sinus congestion	344 (33.4%)	0.73 (0.55-0.98)	*p* = 0.03*
clear nasal discharge	215 (20.9%)	0.71 (0.50-0.99)	*p* = 0.04*
purulent nasal discharge	184 (17.8%)	0.83 (0.58-1.19)	p = 0.33
post-nasal drip sensation	390 (37.8%)	0.93 (0.70-1.21)	*p* = 0.59
sinus pain	79 (7.7%)	1.69 (1.05-2.69)	*p* = 0.03*
sneezing	101 (9.8%)	0.39 (0.22-0.66)	*p* < 0.001**
pulmonary symptoms			
sleep disruption due to cough	610 (59.2%)	1.22 (0.93-1.59)	*p* = 0.15
sputum production	638 (61.9%)	0.98 (0.75-1.29)	*p* = 0.91
shortness of breath	348 (33.8%)	0.74 (0.56-0.97)	*p* = 0.03*
chest tightness	277 (26.8%)	0.94 (0.69-1.26)	*p* = 0.67
wheezing	275 (26.7%)	0.64 (0.48-0.86)	*p* = 0.003*
chest pain	232 (22.5%)	0.82 (0.60-1.12)	*p* = 0.22
paroxysms of cough	315 (30.6%)	0.98 (0.73-1.30)	*p* = 0.87
ear symptoms			
ear pain	48 (4.7%)	1.02 (0.53-1.88)	*p* = 0.94
ear pressure sensation	122 (11.8%)	0.89 (0.58-1.35)	*p* = 0.60
decreased hearing	34 (3.3%)	0.81 (0.35-1.69)	*p* = 0.58
GI symptoms			
loss of appetite	129 (12.5%)	0.82 (0.54-1.23)	*p* = 0.34
abdominal pain	25 (2.4%)	0.30 (0.07-0.87)	*p* = 0.05
post-tussive vomiting	74 (7.2%)	0.67 (0.41-1.09)	*p* = 0.10
nausea	63 (6.1%)	0.46 (0.22-0.86)	*p* = 0.02*
vomiting	48 (4.6%)	0.44 (0.19-0.89)	*p* = 0.03*
diarrhea	34 (3.3%)	1.24 (0.59-2.49)	*p* = 0.56
neuro-vascular symptoms			
lightheadedness	47 (4.6%)	1.29 (0.69-2.35)	*p* = 0.41
Commonly Ordered Labs			
chest x-ray	177 (17.2%)	2.09 (1.50-2.90)	*p* < 0.001**
rapid strep screen	31 (3.0%)	0.65 (0.26-1.45)	*p* = 0.32
complete blood count	85 (8.2%)	1.40 (0.88-2.21)	*p* = 0.15
Monospot	27 (2.6%)	1.83 (0.83-3.95)	*p* = 0.12
influenza A + B	16 (1.6%)	0.32 (0.05-1.14)	*p* = 0.13
Common Exam Findings†			
ear exam			
tympanic membrane (TM)	27 (2.6%)	0.08 (0.004-0.40)	*p* = 0.01*
bulging			
TM retraction	42 (4.1%)	0.79 (0.38-1.55)	*p* = 0.51
visible fluid behind TM	148 (14.3%)	0.17 (0.09-0.29)	*p* < 0.001**
cerumen in canal	29 (2.8%)	0.46 (0.15-1.12)	*p* = 0.12
nose exam			
mucosal edema	580 (56.2%)	0.83 (0.63-1.08)	*p* = 0.16
mucosal erythema	510 (49.5%)	0.78 (0.59-1.01)	*p* = 0.06
nasal discharge	324 (31.4%)	1.59 (1.20-2.09)	*p* = 0.001**
maxillary sinus tenderness	30 (2.9%)	1.75 (0.83-3.64)	*p* = 0.13
throat exam			
erythema	209 (20.3%)	0.79 (0.56-1.10)	*p* = 0.17
lymphoid hyperplasia	104 (10.1%)	1.47 (0.96-2.22)	*p* = 0.07
post-nasal drip	157 (15.2%)	1.44 (1.00-2.04)	*p* = 0.04*
tonsil exam			
surgically absent	42 (4.1%)	1.13 (0.57-2.14)	*p* = 0.71
erythema	84 (8.1%)	0.43 (0.23-0.74)	*p* = 0.004**
enlarged	47 (4.5%)	1.17 (0.62-2.32)	*p* = 0.64
lymphatics exam			
anterior cervical lymphadenopathy, tender	53 (5.1%)	1.93 (1.10-3.38)	*p* = 0.02*
anterior cervical lymphadenopathy, non-tender	87 (8.4%)	0.70 (0.41-1.14)	*p* = 0.16
posterior cervical lymphadenopathy, non-tender	51 (4.9%)	0.60 (0.29-1.16)	*p* = 0.15
anterior cervical lymphadenopathy	99 (9.6%)	3.05 (2.01-4.66)	*p* < 0.001**
posterior cervical lymphadenopathy	26 (2.5%)	1.00 (0.41-2.25)	*p* = 0.10
lung exam			
wheezing	215 (20.9%)	1.92 (1.40-2.61)	*p* < 0.001**
rales	21 (2.0%)	10.05 (3.69-35.18)	*p* < 0.001**
rhonchi	223 (21.6%)	2.33 (1.71-3.16)	*p* < 0.001**
percussion abnormality	25 (2.4%)	9.55 (3.83-28.91)	*p* < 0.001**

**Fig. 1:**
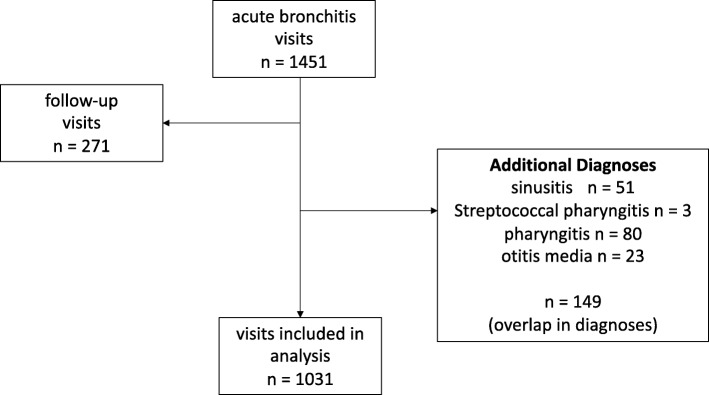
Flow of study inclusion and exclusion criteria for acute bronchitis visits (*n* = number of visits). Side arrows indicate exclusion criteria

We subsequently excluded data on 271 follow-up visits within UHS for previously diagnosed conditions and 149 visits with additional diagnoses for which antibiotics might be appropriate (sinusitis, pharyngitis, streptococcal pharyngitis, otitis media). One thousand thirty-one visits were included in the final analysis (Fig. 1).

Data from electronic patient charts included variables for all symptoms and exam findings listed in the record system. Many of these symptoms (e.g. eye discharge, mouth sores) were uncommon in acute bronchitis visits. To narrow the list to variables that might be important in acute bronchitis visits, as well as to eliminate variables with zero frequency cells in univariate contingency tables, we only considered symptoms and exam findings recorded for > 20 patients for subsequent analysis (Table 1).

Four visits had onset durations that were extreme outliers (> 100 days since onset), and we substituted missing values for these onset durations. Models excluded visits with missing values in predictor variables. This strategy resulted in 33 visits being excluded from analysis in the final multivariate model due to missing values in predictor variables. It is important to note that for two patient-reported variables, severity and progression, “not recorded” was coded as a factor level, and these entries were not considered missing values.

### Statistical methods

In all analyses, the response variable was whether an antibiotic was prescribed at a visit. All variables listed in Table 1 were tested as possible predictive factors. Bivariate logistic regression analyses were used to identify a narrowed list of potential predictors of antibiotic prescribing (Table 1) [35]. Provider traits were not included in the logistic regression analysis due to the small number of providers in the data set (21 total). All variables identified as significant in the bivariate analyses were entered into multivariate logistic regression analyses to identify independent predictors of antibiotic prescribing for acute bronchitis. Backward stepwise removal of nonsignificant variables was used to generate the final multivariate model [35]. Factors were considered significant in the regression analyses when they had *p*-values < 0.05. Analyses were carried out using R (version 3.4.3).

## Results

### Study population and antibiotic prescribing

**Fig. 2:**
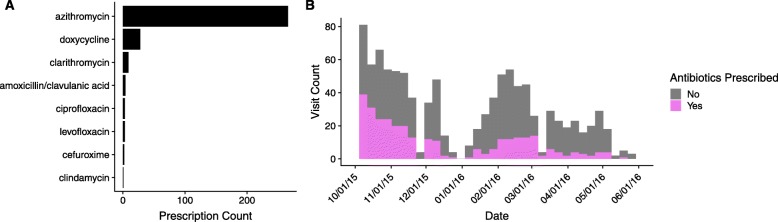
Antibiotic prescribing. **a** Antibiotic prescriptions by drug. **b** Visits and antibiotic prescribing over time

The data set included 1031 visits with an acute bronchitis diagnosis (Table 1). 61.7% of patients were female, and 90.1% of patients were undergraduate students. Antibiotics were prescribed at 30.8% of visits. Azithromycin was the most commonly prescribed antibiotic (83.9% of prescriptions) (Fig. 2a). Figure 2b shows the distribution of acute bronchitis visits and rates of antibiotic prescribing over the course of the 2015–2016 academic year. Table 1 shows the frequency of antibiotic prescribing by variable.

### Factors associated with antibiotic use

**Fig. 3:**
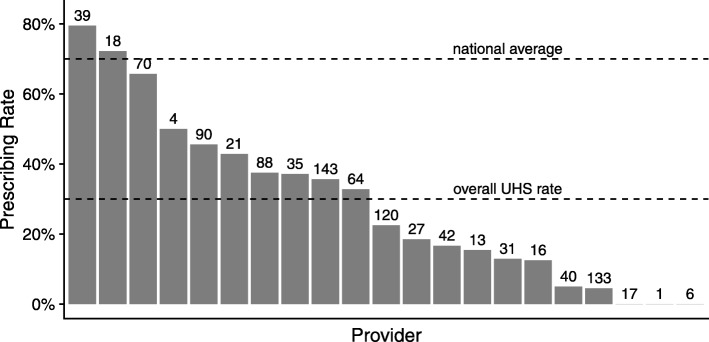
Antibiotic prescribing rates by provider. Prescribing rates for acute bronchitis visits were highly variable among providers. The total number of acute bronchitis visits for each provider is shown above the bar, together with the national average [7–9, 16], and the overall rate at the PSU health facility

Factors independently associated with antibiotic prescribing in a multivariate regression model are summarized in Table 2. The factors with the greatest impacts on prescribing were individual providers and suspicious findings during lung examinations. The 21 providers in the data set had dramatically different rates of prescribing for acute bronchitis cases ranging from 0 to 80% (Fig. 3), and provider was an important predictor of prescribing (odds ratios (OR) ranged from 0.03 to 12.3 for individual providers). Suspicious findings during lung examinations were highly associated with antibiotic prescribing (rales OR 13.95, 95% CI 3.31–80.73; rhonchi OR 5.50, 95% CI 3.08–10.00; percussion abnormality OR 13.02, 95% CI 4.00–50.09).

**Table 2:** Factors independently associated with prescribing in a multivariate model

Variable	Odds Ratio (95% CI)	*p*-value
Visit and Patient Characteristics		
visit date (days)	0.99 (0.99-0.99)	*p* < 0.001**
gender		
female (reference group)		
male	1.68 (1.17-2.41)	*p* = 0.005**
provider	0.03-12.3	*p* < 0.001**
onset duration (days)	1.04 (1.03-1.06)	*p* < 0.001**
progression		
stable/no change (reference group)		
worsening	1.78 (1.03-3.10)	*p* = 0.04*
improving	0.74 (0.35-1.54)	*p* = 0.43
not recorded	1.69 (0.87-3.27)	*p* = 0.12
Additional Diagnosis		
URI diagnosis	0.33 (0.18-0.58)	*p* < 0.001**
Symptoms		
sneezing	0.39 (0.17-0.86)	*p* = 0.02*
vomiting	0.31 (0.10-0.83)	*p* = 0.03*
Exam Findings		
lymphatics		
anterior cervical lymphadenopathy, tender	3.85 (1.70-8.83)	*p* = 0.001**
anterior cervical lymphadenopathy	2.63 (1.25-5.54)	*p* = 0.01*
lungs		
rales	13.95 (3.31-80.73)	*p* = 0.001**
rhonchi	5.50 (3.08-10.00)	*p* < 0.001**
percussion abnormality	13.02 (4.00-50.09)	*p* < 0.001**

The model showed that prescribing rates decreased slightly over the course of the academic year (OR 0.99 per day, 95% CI 0.99–0.99). Male patients were more likely than female patients to be prescribed antibiotics (OR 1.68, 95% CI 1.17–2.41). Patients who reported longer duration since the onset of symptoms were slightly more likely to receive prescriptions (OR 1.04 per day, 95% CI 1.03–1.06), as were patients who reported their symptoms were worsening (OR 1.78, 95% CI 1.03–3.10). Visits with additional diagnoses or symptoms associated with viral infections or allergies were less likely to result in prescriptions (URI diagnosis OR 0.33, 95% CI 0.18–0.58; sneezing OR 0.39, 95% CI 0.17–0.86; vomiting OR 0.31, 95% CI 0.10–0.83). An exam finding of anterior cervical lymphadenopathy was associated with antibiotic prescribing (tender OR 3.85, 95% CI 1.70–8.83; general OR 2.63, 95% CI 1.25–5.54).

**Fig. 4:**
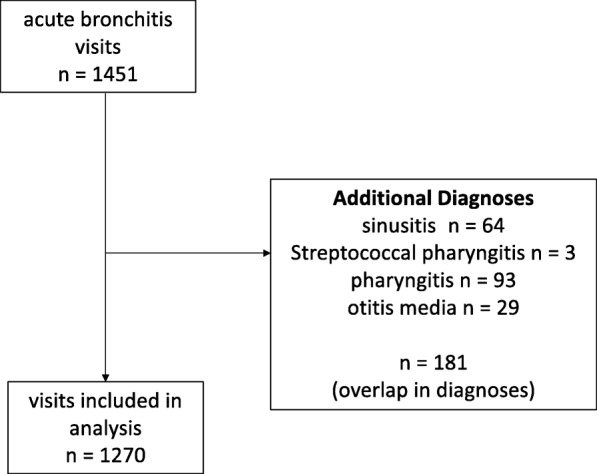
Flow of study inclusion and exclusion criteria for acute bronchitis visits (*n* = number of visits). Side arrows indicate exclusion criteria

As a check, we repeated these analyses without excluding the data from follow-up visits (*N* = 149) for previously diagnosed conditions (Figure 4 in Appendix). The results of this analysis were qualitatively similar to the primary analysis, with the addition of antibiotic prescriptions in the past month as a predictor of prescribing (Tables 3 and 4 in Appendix). Patients who reported taking antibiotics in the past month were less likely to be prescribed antibiotics (OR 0.31, 95% CI 0.14–0.66). Provider and lung exam findings were the strongest predictors of prescribing in both analyses. Visit date, duration since onset, progression, URI diagnosis, sneezing, and anterior cervical lymphadenopathy were also significant predictors in both analyses.

## Discussion

This study’s results indicated two key drivers of antibiotic prescribing: variation between individual providers and diagnostic uncertainty. We take each of these in turn. Individual providers had extraordinarily variable rates of antibiotic prescribing for acute bronchitis (ranging from 0 to 80%), despite treating the same patient population at the same clinic. These results suggest that a subset of providers can drive a disproportionate amount of unnecessary antibiotic prescribing for acute bronchitis. In the current study, provider traits (e.g. provider specialty, age) were not included in the logistic regression analysis due to the small number of providers in the data set (21 total). Previous studies have identified provider specialty, provider age, and perceived patient demand for antibiotics as factors influencing provider prescribing rates for upper respiratory tract infections [9, 20, 26, 36, 37].

A second important driver may be diagnostic uncertainty. In the present study, prescriptions were much more likely when findings of rales, rhonchi, or percussion abnormalities were recorded during lung examination, and somewhat more likely when external anterior cervical lymphadenopathy was reported. Rales and percussion abnormalities increased prescribing 13-fold, and rhonchi increased prescribing 5-fold. This increase in prescribing may reflect suspicion of pneumonia. Orders of chest x-rays, which also indicate suspicion of pneumonia, were a significant predictor of prescribing in a bivariate analysis, but were not significant in a multivariate model due to high correlation with other lung exam findings. Providers may prescribe antibiotics when there is suspicion of a condition that would respond to antibiotics or general diagnostic uncertainty [27], and this may not be reflected in the diagnosis code.

Other predictors of prescribing in this study included symptoms of sneezing and vomiting, reported worsening of symptoms, diagnosis of an upper respiratory tract infection, duration of illness, and patient gender. Duration of illness has previously been associated with prescribing for upper respiratory tract infections [28]. Patient gender has not typically been associated with prescribing rates for acute bronchitis [16, 20, 28, 29], although some studies have reported that males are more likely to get antibiotic prescriptions for upper respiratory tract infections [8, 17].

The identification of provider variation and diagnostic uncertainty as drivers of prescribing suggests possible interventions for this clinic and similar settings. Provider variation points to a need for provider-targeted interventions such as audit and feedback, communication training, provider education, or clinical decision support tools [22, 23]. In an ‘audit and feedback’ intervention, individual clinicians receive personalized, ongoing feedback on their prescribing rates [22–24, 38, 39]. In one study, quarterly feedback resulted in a 50% relative reduction in broad-spectrum antibiotic use for respiratory tract infections [24]. Provider communication training has also been shown to decrease unnecessary antibiotic prescribing [23]. Communication training addresses provider concerns related to patient satisfaction and patient expectation for antibiotics [23]. In some cases, diagnostic uncertainty may be addressed through point of care diagnostic testing [22]. Point of care diagnostics are available for respiratory tract infections including Group A *Streptococcus* and influenza [22]. There is some evidence supporting point of care testing to reduce antibiotic prescribing for respiratory tract infections [23, 40].

While unnecessary prescribing for acute bronchitis was common in our data, the rate of prescribing was substantially lower than the nationwide average. In the 2015–2016 academic year, antibiotics were prescribed at less than a third of acute bronchitis visits, compared to national rates near 70% [7–9, 16]. There is still room for improvement, but overall, this suggests that lower rates of prescribing for acute bronchitis are achievable.

Our study is unique in its focus on antibiotic prescribing practices at a university health center. University health services are important centers for antibiotic prescribing serving millions of patients, yet they have largely been overlooked as sites for antibiotic stewardship. To our knowledge, the Pennsylvania State University is the first university with a student antibiotic stewardship program. This study is the first to identify drivers of antibiotic prescribing in a university health center, and one of the few to focus on young adults or consider exam findings and symptoms from patient charts as possible predictors of prescribing. We hope that these findings can be used to inform antibiotic stewardship initiatives at university health centers and similar clinical contexts. Our results suggest that unnecessary antibiotic prescribing is disproportionately driven by a subset of clinicians, and interventions targeting providers may be effective at reducing unnecessary prescribing.

## Conclusions

Reducing unnecessary antibiotic prescribing for acute bronchitis cases is a national antibiotic stewardship goal, yet rates of unnecessary antibiotic prescribing remain stubbornly high nationwide. Here we identified factors that influence antibiotic prescribing for acute bronchitis cases at a large university health center. Suspicious findings during lung examinations (rales, rhonchi, percussion abnormality) and individual healthcare providers were the most influential factors affecting antibiotic prescribing rates for acute bronchitis visits. Patient gender, worsening symptoms, duration of illness, symptoms associated with viral infections or allergies, and anterior cervical lymphadenopathy also influenced prescribing rates.

## Data Availability

The datasets analyzed during the current study are available from the corresponding author on reasonable request.

## Appendix

### Analysis including follow-up visits

The logistic regression analyses described in the main text were repeated with a data set including follow-up visits for previously diagnosed conditions, which had been excluded from the original analysis. Antibiotics were prescribed at 30.0% of visits.

**Table 3:** Descriptive statistics (*n* = 1270) and bivariate analysis

Variable	Visits	Odds Ratio (95% CI)	Bivariate*p*-value
Date and Time			
visit date	1267 (99.8%)	0.99 (0.99-0.99)	*p* < 0.001 **
week day	1267 (99.8%)	range 0.60-1.40	*p* = 0.26
time of day	1258 (99.0%)	0.99 (0.99-1.00)	*p* = 0.61
Patient Characteristics			
gender			*p* = 0.07
female (reference group)	788 (62.0%)	-	
male	477 (46.3%)	1.25 (0.98-1.60)	
not recorded	5 (0.4%)	-	
race			*p* = 0.20
white (reference group)	737 (58.0%)	-	
multiple	117 (9.2%)	0.76 (0.48-1.17)	
Asian	61 (4.8%)	0.88 (0.49-1.54)	
black	31 (2.4%)	1.00 (0.45-2.11)	
Hispanic	7 (0.6%)	-	
international	8 (0.6%)	-	
Pacific islander	2 (0.1%)	-	
not recorded	307 (24.2%)	-	
academic status			*p* = 0.70
undergraduate student (reference group)	1153 (90.8%)	-	
graduate student	101 (7.9%)	0.83 (0.52-1.30)	
spouse/dependent	4 (0.3%)	-	
not recorded	12 (0.9%)	-	
height (inches)	1242 (97.8%)	1.03 (1.00-1.07)	*p* = 0.04*
weight (pounds)	1236 (97.3%)	1.00 (0.99-1.01)	*p* = 0.15
Visit Characteristics			
provider	1254 (98.7%)	range 0.07 -3.27	*p* < 0.001**
onset duration (patient reported)	1250 (98.4%)	0.99 (0.99-1.00)	*p* < 0.001**
severity (patient reported)			*p* = 0.02*
mild (reference group)	69 (5.4%)	-	
moderate	381 (30.0%)	0.54 (0.32-0.95)	
severe	37 (2.9%)	1.20 (0.52-2.72)	
not recorded	783 (61.6%)	-	
timing (patient reported)			*p* < 0.001**
stable/no change (reference group)	322 (25.3%)	-	
worsening	370 (29.1%)	2.59 (1.84-3.65)	
improving	176 (13.8%)	0.67 (0.40-1.09)	
not recorded	402 (31.6%)	-	
antibiotics in past month	85 (6.7%)	0.44 (0.23-0.77)	*p* = 0.006**
Additional Diagnosis			
URI	216 (17.0%)	0.30 (0.19-0.44)	*p* < 0.001**
suspicious cough	102 (8.0%)	1.30 (0.84-1.98)	*p* = 0.22
allergic rhinitis	38 (3.0%)	0.43 (0.16-0.96)	*p* = 0.06
fever	19 (1.5%)	2.13 (0.84-5.31)	*p* = 0.10
viral syndrome	15 (1.2%)	-	-
tonsillitis	6 (0.4%)	-	-
influenza	5 (0.4%)	-	-
mononucleosis	7 (0.5%)	-	-
Common Symptoms Recorded†			
throat symptoms			
sore throat	459 (36.1%)	1.45 (1.13-1.86)	*p* = 0.003**
painful swallowing	146 (11.5%)	0.60 (0.39-0.89)	*p* = 0.01*
hoarseness	186 (14.6%)	0.84 (0.59-1.18)	*p* = 0.32
swollen glands in neck	126 (9.9%)	1.19 (0.80-1.75)	*p* = 0.39
systemic symptoms			
headache	272 (21.4%)	0.88 (0.65-1.18)	*p* = 0.40
documented fever	73 (5.7%)	0.63 (0.39-1.04)	*p* = 0.06
fever symptoms (patient reported)	285 (22.4%)	1.10 (0.83-1.46)	*p* = 0.51
chills	164 (12.9%)	0.86 (0.60-1.22)	*p* = 0.38
sweats	182 (14.3%)	1.02 (0.73-1.45)	*p* = 0.92
nasal symptoms			
stuffy nose	782 (61.6%)	1.04 (0.81-1.33)	*p* = 0.76
sinus congestion	402 (39.0%)	0.83 (0.64-1.08)	*p* = 0.16
clear nasal discharge	251 (19.8%)	0.86 (0.63-1.16)	*p* = 0.33
purulent nasal discharge	212 (16.7%)	0.96 (0.69-1.32)	*p* = 0.79
post-nasal drip sensation	444 (35.0%)	1.10 (0.86-1.41)	*p* = 0.46
sinus pain	85 (6.7%)	1.79 (1.14-2.79)	*p* = 0.01*
sneezing	108 (8.5%)	0.47 (0.28-0.77)	*p* = 0.004**
pulmonary symptoms			
sleep disruption due to cough	710 (55.9%)	1.08 (0.85-1.37)	*p* = 0.54
sputum production	743 (58.5%)	0.94 (0.73-1.20)	*p* = 0.61
shortness of breath	412 (32.4%)	0.69 (0.53-0.88)	*p* = 0.003**
chest tightness	331 (26.1%)	0.82 (0.62-1.07)	*p* = 0.14
wheezing	340 (26.8%)	0.64 (0.49-0.84)	*p* = 0.001**
chest pain	269 (21.2%)	0.75 (0.56-0.99)	*p* = 0.047*
paroxysms of cough	362 (28.5%)	0.91 (0.70-1.18)	*p* = 0.46
ear symptoms			
ear pain	59 (4.6%)	1.03 (0.57-1.78)	*p* = 0.93
ear pressure sensation	143 (11.2%)	0.93 (0.63-1.36)	*p* = 0.71
decreased hearing	41 (3.2%)	0.96 (0.47-1.87)	*p* = 0.92
GI symptoms			
loss of appetite	146 (11.5%)	0.80 (0.53-1.17)	*p* = 0.26
abdominal pain	29 (2.3%)	0.26 (0.06-0.75)	*p* = 0.03*
post-tussive vomiting	85 (6.7%)	0.59 (0.38-0.92)	*p* = 0.02*
nausea	72 (5.7%)	0.50 (0.26-0.89)	*p* = 0.02*
vomiting	58 (4.6%)	0.73 (0.38-1.32)	*p* = 0.32
diarrhea	37 (2.9%)	1.27 (0.62-2.49)	*p* = 0.49
neuro-vascular symptoms			
lightheadedness	59 (4.6%)	1.30 (0.74-2.24)	*p* = 0.33
Commonly Ordered Labs			
chest x-ray	223 (17.6%)	2.17 (1.61-2.91)	*p* < 0.001**
rapid strep screen	37 (2.9%)	0.54 (0.21-1.16)	*p* = 0.14
complete blood count	115 (9.0%)	1.38 (0.92-2.06)	*p* = 0.11
monospot	36 (2.8%)	1.33 (0.65-2.61)	*p* = 0.41
influenza A + B	19 (1.5%)	0.27 (0.04-0.95)	*p* = 0.08
Common Exam Findings†			
ear exam			
tympanic membrane (TM) bulging	32 (2.5%)	0.07 (0.004-0.34)	*p* = 0.01*
TM retraction	48 (3.8%)	0.77 (0.38-1.46)	*p* = 0.44
visible fluid behind TM	173 (13.6%)	0.18 (0.10-0.30)	*p* < 0.001**
cerumen in canal	37 (2.9%)	0.64 (0.27-1.34)	*p* = 0.26
nose exam			
mucosal edema	707 (55.7%)	0.83 (0.65-1.06)	*p* = 0.14
mucosal erythema	606 (47.7%)	0.80 (0.63-1.02)	*p* = 0.07
nasal discharge	382 (30.1%)	1.58 (1.22-2.04)	*p* < 0.001**
maxillary sinus tenderness	35 (2.7%)	2.01 (1.01-3.95)	*p* = 0.04*
throat exam			
erythema	240 (18.9%)	0.76 (0.55-1.04)	*p* = 0.09
lymphoid hyperplasia	114 (9.0%)	1.59 (1.07-2.36)	*p* = 0.02*
post-nasal drip	181 (14.2%)	1.41 (1.01-1.95)	*p* = 0.04*
tonsil exam			
surgically absent	56 (4.4%)	0.93 (0.50-1.65)	*p* = 0.81
erythema	97 (7.6%)	0.47 (0.27-0.79)	*p* = 0.006**
enlarged	57 (4.5%)	1.10 (0.62-2.04)	*p* = 0.74
lymphatics exam			
anterior cervical lymphadenopathy, tender	64 (5.0%)	1.88 (1.12-3.12)	*p* = 0.01*
anterior cervical lymphadenopathy, non-tender	103 (8.1%)	0.81 (0.51-1.27)	*p* = 0.38
posterior cervical lymphadenopathy, non-tender	62 (4.9%)	0.67 (0.35-1.20)	*p* = 0.19
anterior cervical lymphadenopathy	132 (10.4%)	2.80 (1.94-4.04)	*p* < 0.001**
posterior cervical lymphadenopathy	38 (3.0%)	1.08 (0.52-2.12)	*p* = 0.83
lung exam			
wheezing	253 (19.9%)	1.82 (1.37-2.43)	*p* < 0.001**
rales	22 (1.7%)	8.26 (3.24-25.27)	*p* < 0.001**
rhonchi	273 (21.5%)	2.30 (1.74-3.03)	*p* < 0.001**
percussion abnormality	30 (2.4%)	9.89 (4.27-26.89)	*p* < 0.001**

^†^ includes symptoms and findings recorded for > 20 visits

**Table 4:** Factors independently associated with prescribing in a multivariate model

Variable	Odds Ratio (95% CI)	p-value
Visit and Patient Characteristics		
visit date (days)	0.99 (0.99-0.99)	*p* < 0.001**
height (inches)	1.05 (1.00-1.09)	*p* = 0.03*
provider	range 0.04-8.42	*p* < 0.001**
onset duration (days)	1.04 (1.03-1.05)	*p* < 0.001**
progression		
stable/no change (reference group)		
worsening	1.79 (1.11-2.90)	p = 0.02*
improving	0.43 (0.23-0.81)	*p* = 0.01*
not recorded	1.43 (0.83-2.48)	*p* = 0.20
antibiotics in past month	0.32 (0.14-0.65)	*p* = 0.003**
Diagnosis		
URI diagnosis	0.36 (0.21-0.62)	*p* < 0.001**
Symptoms		
sore throat	1.46 (1.04-2.05)	*p* = 0.03*
sneezing	0.48 (0.22-0.97)	*p* = 0.048*
Exam Findings		
lymphatics		
anterior cervical lymphadenopathy, tender	2.55 (1.27-5.13)	*p* = 0.008**
anterior cervical lymphadenopathy	2.88 (1.55- 5.39)	*p* < 0.001**
lungs		
rales	10.21 (3.16-60.02)	*p* < 0.001**
rhonchi	5.08 (3.10-8.49)	*p* < 0.001**
percussion abnormality	9.69 (3.47-30.79)	*p* < 0.001**

## Abbreviations

GI: Gastrointestinal
PSU: Pennsylvania State University
TM: Tympanic membrane
UHS: University Health Services
URI: Upper respiratory tract infection

## References

[1] Fleming-Dutra KE, Hersh AL, Shapiro DJ (2016). Prevalence of inappropriate antibiotic prescriptions among U.S. ambulatory care visits, 2010-2011. JAMA..

[2] Pew Research Center. Antibiotic use in outpatient settings. Washington D.C: The Pew Chartible Trusts; 2016.

[3] Friedman ND, Temkin E, Carmeli Y (2016). The negative impact of antibiotic resistance. Clin Microbiol Infect.

[4] Holmes AH, Moore LSP, Sundsfjord A, Steinbakk M, Regmi S, Karkey A (2016). Understanding the mechanisms and drivers of antimicrobial resistance. Lancet..

[5] zur Wiesch PA, Kouyos R, Engelstädter J, Regoes RR, Bonhoeffer S (2011). Population biological principles of drug-resistance evolution in infectious diseases. Lancet Infect Dis.

[6] The White House (2015). National action plan for combating antibiotic-resistant bacteria.

[7] Barnett ML, Linder JA (2014). Antibiotic prescribing for adults with acute bronchitis in the United States, 1996-2010. JAMA..

[8] Brown DW, Taylor R, Rogers A, Weiser R, Kelley M (2003). Antibiotic prescriptions associated with outpatient visits for acute upper respiratory tract infections among adult Medicaid recipients in North Carolina. N C Med J.

[9] Schmidt ML, Spencer MD, Davidson LE (2018). Patient, provider, and practice characteristics associated with inappropriate antimicrobial prescribing in ambulatory practices. Infect Control Hosp Epidemiol.

[10] Gonzales R, Steiner JF, Sande MA (1997). Antibiotic prescribing for adults with colds, upper respiratory tract infections, and bronchitis by ambulatory care physicians. JAMA J Am Med Assoc.

[11] Smith SM, Fahey T, Smucny J, Becker LA (2017). Antibiotics for acute bronchitis. Cochrane Database Syst Rev.

[12] Talwalkar A, Hing E, Palso K (2011). National Hospital Ambulatory Medical Care Survey: 2011 outpatient department summary tables.

[13] Macfarlane J, Holmes W, Gard P, Macfarlane R, Rose D, Weston V (2001). Prospective study of the incidence, aetiology and outcome of adult lower respiratory tract illness in the community. Thorax..

[14] Gonzales R, Bartlett JG, Besser RE, Cooper RJ, Hickner JM, Hoffman JR (2001). Principles of appropriate antibiotic use for treatment of uncomplicated acute bronchitis: background. Ann Intern Med.

[15] Irwin RS, Baumann MH, Bolser DC, Boulet L-P, Braman SS, Brightling CE (2006). Diagnosis and management of cough executive summary. Chest..

[16] Grigoryan L, Zoorob R, Shah J, Wang H, Arya M, Trautner BW (2017). Antibiotic prescribing for uncomplicated acute bronchitis is highest in younger adults. Antibiotics..

[17] Xu KT, Roberts D, Sulapas I, Martinez O, Berk J, Baldwin J (2013). Over-prescribing of antibiotics and imaging in the management of uncomplicated URIs in emergency departments. BMC Emerg Med.

[18] Aspinall SL, Good CB, Metlay JP, Mor MK, Fine MJ (2009). Antibiotic prescribing for presumed nonbacterial acute respiratory tract infections. Am J Emerg Med.

[19] Donnelly JP, Baddley JW, Wang HE (2014). Antibiotic utilization for acute respiratory tract infections in U.S. emergency departments. Antimicrob Agents Chemother.

[20] McKay R, Mah A, Law MR, McGrail K, Patrick DM (2016). Systematic review of factors associated with antibiotic prescribing for respiratory tract infections. Antimicrob Agents Chemother.

[21] Antibiotic use in the United States (2019). 2018 Update: Progress and opportunities.

[22] Dobson EL, Klepser ME, Pogue JM, Labreche MJ, Adams AJ, Gauthier TP (2017). Outpatient antibiotic stewardship: interventions and opportunities. J Am Pharm Assoc.

[23] Drekonja DM, Filice GA, Greer N, Olson A, MacDonald R, Rutks I (2015). Antimicrobial stewardship in outpatient settings: a systematic review. Infect Control Hosp Epidemiol.

[24] Gerber JS, Prasad PA, Fiks AG, Localio AR, Grundmeier RW, Bell LM (2013). Effect of an outpatient antimicrobial stewardship intervention on broad-spectrum antibiotic prescribing by primary care pediatricians a randomized trial. JAMA.

[25] Avorn J, Solomon DH (2000). Cultural and economic factors that (mis) shape antibiotic use: the nonpharmacologic basis of therapeutics. Ann Intern Med.

[26] O’Connor R, O’Doherty J, O’Regan A, Dunne C (2018). Antibiotic use for acute respiratory tract infections (ARTI) in primary care; what factors affect prescribing and why is it important? A narrative review. Ir J Med Sci.

[27] Whaley LE, Businger AC, Dempsey PP, Linder JA (2013). Visit complexity, diagnostic uncertainty, and antibiotic prescribing for acute cough in primary care: a retrospective study. BMC Fam Pract.

[28] Gonzales R, Camargo CA, MacKenzie T, Kersey AS, Maselli J, Levin SK (2006). Antibiotic treatment of acute respiratory infections in acute care settings. Acad Emerg Med.

[29] Gonzales R, Barrett PH, Crane LA, Steiner JF, Steiner JF (1998). Factors associated with antibiotic use for acute bronchitis. J Gen Intern Med.

[30] Snyder TD, de Brey C, Dillow S (2019). Digest of education statistics: 2017.

[31] Turner JC, Keller A (2015). College health surveillance network: epidemiology and health care utilization of college students at us 4-year universities. J Am Coll Heal.

[32] Atkinson I (2012). Accuracy of data transfer: double data entry and estimating levels of error. J Clin Nurs.

[33] Day S, Fayers P, Harvey D (1998). Double data entry: what value, what price?. Control Clin Trials.

[34] Barchard KA, Pace LA (2011). Preventing human error: the impact of data entry methods on data accuracy and statistical results. Comput Human Behav.

[35] Hosmer David W., Lemeshow Stanley, Sturdivant Rodney X. (2013). Applied Logistic Regression.

[36] Zuckerman IH, Perencevich EN, Harris AD (2007). Concurrent acute illness and comorbid conditions poorly predict antibiotic use in upper respiratory tract infections: a cross-sectional analysis. BMC Infect Dis.

[37] Dempsey PP, Businger AC, Whaley LE, Gagne JJ, Linder JA (2014). Primary care clinicians’ perceptions about antibiotic prescribing for acute bronchitis: a qualitative study. BMC Fam Pract.

[38] Meeker D, Linder JA, Fox CR, Friedberg MW, Persell SD, Goldstein NJ (2016). Effect of behavioral interventions on inappropriate antibiotic prescribing among primary care practices a randomized clinical trial. J Am Med Assoc.

[39] Hemkens LG, Saccilotto R, Reyes SL, Glinz D, Zumbrunn T, Grolimund O (2017). Personalized prescription feedback using routinely collected data to reduce antibiotic use in primary care a randomized clinical trial. JAMA Intern Med.

[40] Klepser Donald G., Klepser Michael E., Dering-Anderson Allison M., Morse Jacqueline A., Smith Jaclyn K., Klepser Stephanie A. (2016). Community pharmacist–physician collaborative streptococcal pharyngitis management program. Journal of the American Pharmacists Association.

